# Geothermal power generation and positive impact in the greater bay area: a case study of Huizhou City, Guangdong Province

**DOI:** 10.1038/s41598-024-53738-1

**Published:** 2024-02-19

**Authors:** Wenquan Cai, Jianying Li, Chuantao Xiao

**Affiliations:** 1 Yangtze University, Wuhan, Hubei China; 2 Guangdong Non-ferrous Metal Geology Bureau, Unit 935, Huizhou, Guangdong China; 3 Guangdong Non-ferrous Metal Geology Bureau, Unit 931, Shantou, Guangdong China

**Keywords:** Environmental sciences, Energy science and technology, Engineering

## Abstract

Geothermal resources, as abundant renewable and clean natural resources on Earth, have been utilized for geothermal power generation in 29 countries. Guangdong Province, as one of the most developed, economically vibrant, and attractive investment regions in China, has experienced rapid economic growth. However, further development is constrained by resource limitations and severe ecological environmental pressures. The issue of energy consumption and economic growth imbalance needs to be urgently addressed. Geothermal energy, as a clean and stable form of energy, can reduce reliance on fossil fuels, decrease dependence on imported energy, and enhance national energy security. In this study, geological survey, geophysical survey, geochemical survey, remote sensing detection and drilling were carried out in the Shiba–Huangshadong area of Huizhou, and by analyzing the data on geothermal energy reserves in the study area, the available geothermal energy and its potentials in the study area were identified and its impact on the current energy consumption in Huizhou City was assessed, to validate the feasibility of the detailed application of the geothermal energy in the Greater Bay Area, which will enable the energy structure of Huizhou City optimize the energy structure.

## Introduction

Geothermal resources, as vast renewable and clean natural resources on Earth, are estimated to have an energy storage of approximately 3.6 × 10^14^ gigawatt-hours in the upper 10 km of the Earth’s crust^1^. Theoretically, these geothermal reserves can supply global energy consumption for approximately 2.17 million years, based on a global energy consumption rate of approximately 1.7 × 10^8^ gigawatt-hours per year in 2012^2,3^. In recent years, geothermal energy has been widely utilized globally, with increasing utilization rates^4^. Currently, most of the geothermal resources being exploited and utilized domestically and internationally are hydrothermal, but other types such as steam, geopressure, hot dry rock, radiogenic, magma and lava, and sedimentary basin types also exist, indicating significant potential for geothermal energy storage and reserves in various forms^5^.

**Figure 1:**
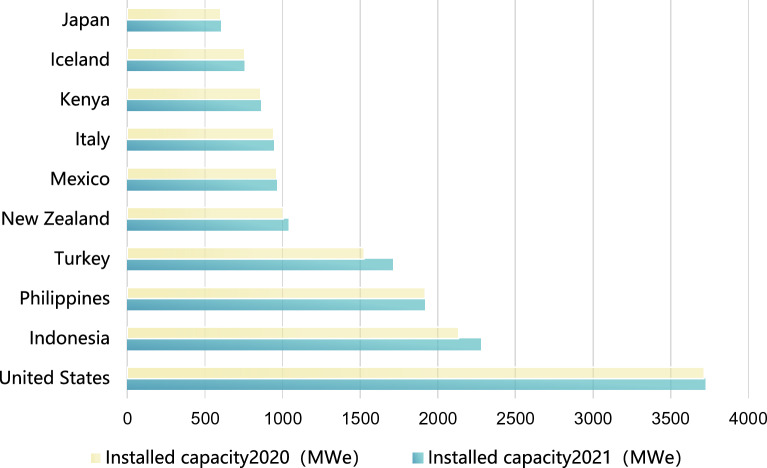
Top 10 countries in geothermal power generation in the world.

In 2021, the global geothermal capacity increased by 246 MW. The total installed capacity of other countries outside the top ten was 1067 MW, bringing the total installed capacity of geothermal power generation to 15,854 MW by the end of 2021. Indonesia had the largest increase, with an additional 143  MW of installed capacity and the construction of two new power plants. This was followed by Chile and Turkey. Currently, there are geothermal power plants in operation in 29 countries/regions^6^.The top ten countries with the highest geothermal power generation capacity are shown in Fig. 1.

China, as the world's largest energy consumer and the second-largest economy, faces challenges of an insufficiently rational energy structure and limited environmental carrying capacity^7^. In this context, geothermal energy holds significant importance as a clean and stable energy source^8^. Additionally, geothermal energy can reduce reliance on fossil fuels, decrease dependence on imported energy, and enhance national energy security^9^. Therefore, the research on the extraction and utilization of geothermal resources in China becomes increasingly urgent^10,11^.

With the implementation of China's reform and opening-up policy in 1978, Guangdong Province in China was among the first to adopt economic reforms, leading to rapid economic growth^12,13^. Even after the changes in epidemic prevention and control policies in 2023, Guangdong Province remains one of the most developed, economically vibrant, and attractive investment regions in China^14^. Despite the epidemic environment, Guangdong's total economic volume reached 12.9 trillion yuan according to the Guangdong Statistical Yearbook (2022), with Shenzhen, as the core engine city in the Guangdong–Hong Kong–Macao Greater Bay Area, fully utilizes its talent advantages to radiate to surrounding regions^15^.

In January 2017, the National Development and Reform Commission, the Ministry of Land and Resources, and the National Energy Administration jointly formulated and released the first special plan for geothermal energy development titled “Thirteenth Five-Year Plan for Geothermal Energy Development and Utilization.” However, as a national strategy, the development and utilization of geothermal energy are still in the early stages, despite rapid technological advancements, and have not yet achieved large-scale application^16^. The overall utilization rate of geothermal resources remains at a relatively low level^17^. Furthermore, the further development of the Greater Bay Area is constrained by resource limitations and severe ecological environmental pressures, with issues of energy consumption and economic growth imbalance in need of urgent resolution^18^.

However, until now, research on geothermal energy has predominantly focused on nationwide or large regional analyses, utilizing broad methodologies that lack detailed insights and regional depth for individual cities or smaller areas. Our study shifts the emphasis to Huizhou City, engaging in data collection within the study area. This approach not only enhances and advances our understanding of geothermal energy utilization but also strategically addresses shortcomings identified in previous research. Concentrating on a smaller, more specific geographic region, our research delves into regional characteristics, employing a comprehensive methodology. The primary objective is to provide recommendations for the utilization of geothermal potential in the Guangdong Province, specifically Huizhou City and the Greater Bay Area, by integrating data collected from two study areas. Through this, we aim to verify the detailed feasibility of geothermal energy application in the Greater Bay Area, ultimately optimizing the region's energy structure.

## Status and context

**Figure 2:**
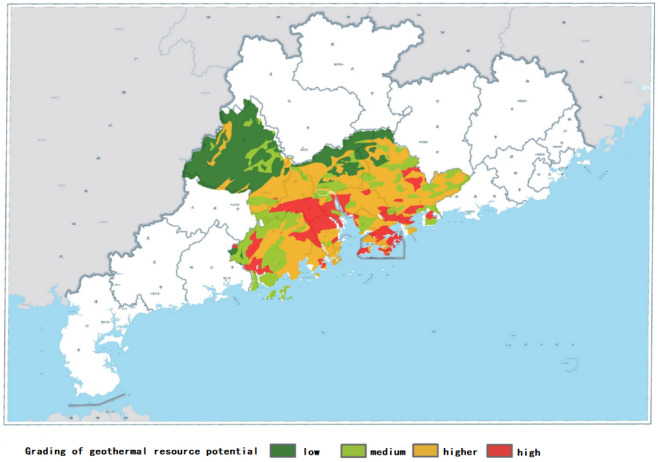
Geothermal resource potential distribution map of Guangdong Province.

Guangdong Province is rich in geothermal energy, and the potential of geothermal resources in the Greater Bay Area is highly rated (Fig. 2). Through investigations, it was found that the geothermal resources in Huizhou City, Guangdong Province, are mainly concentrated in Huidong County and Boluo County. In Huidong County, the resources are primarily distributed in the towns of Fengren, Baihua, Pingshan, Dalin, and Longmen, with Pingshan Town being the most abundant. In Boluo County, the resources are mainly distributed in the towns of Xijiang, Longhua, Yuanzhou, and Luoyang, with Xijiang Town being the most abundant.

However, despite the abundance of geothermal resources (Fig. 2), the current development and utilization methods in Huizhou City, Guangdong Province, remain inefficient. The level of resource exploration is low, and the development and utilization forms are limited to the construction of hot spring tourism as the sole approach^19^.

In order to obtain more detailed data, we selected the Huangsha Cave–Shiba area as the study area and employed various methods for data collection and compilation, including geological investigations, geophysical surveys, geochemical surveys, remote sensing detection, and drilling exploration.

The Huangsha Cave–Shiba area in Huizhou is located at the southern margin of the Cathaysia Block. It is situated within the Meixian-Huiyang sag sedimentary area, bounded by the northeast-trending He Yuan deep fault zone inclined towards the southeast and the northeast-trending Lianhua Mountain deep fault zone inclined towards the northwest (part of the Zhenghe-Dapu Fault southern segment) (Fig. 31a,1b).

**Figure 3:**
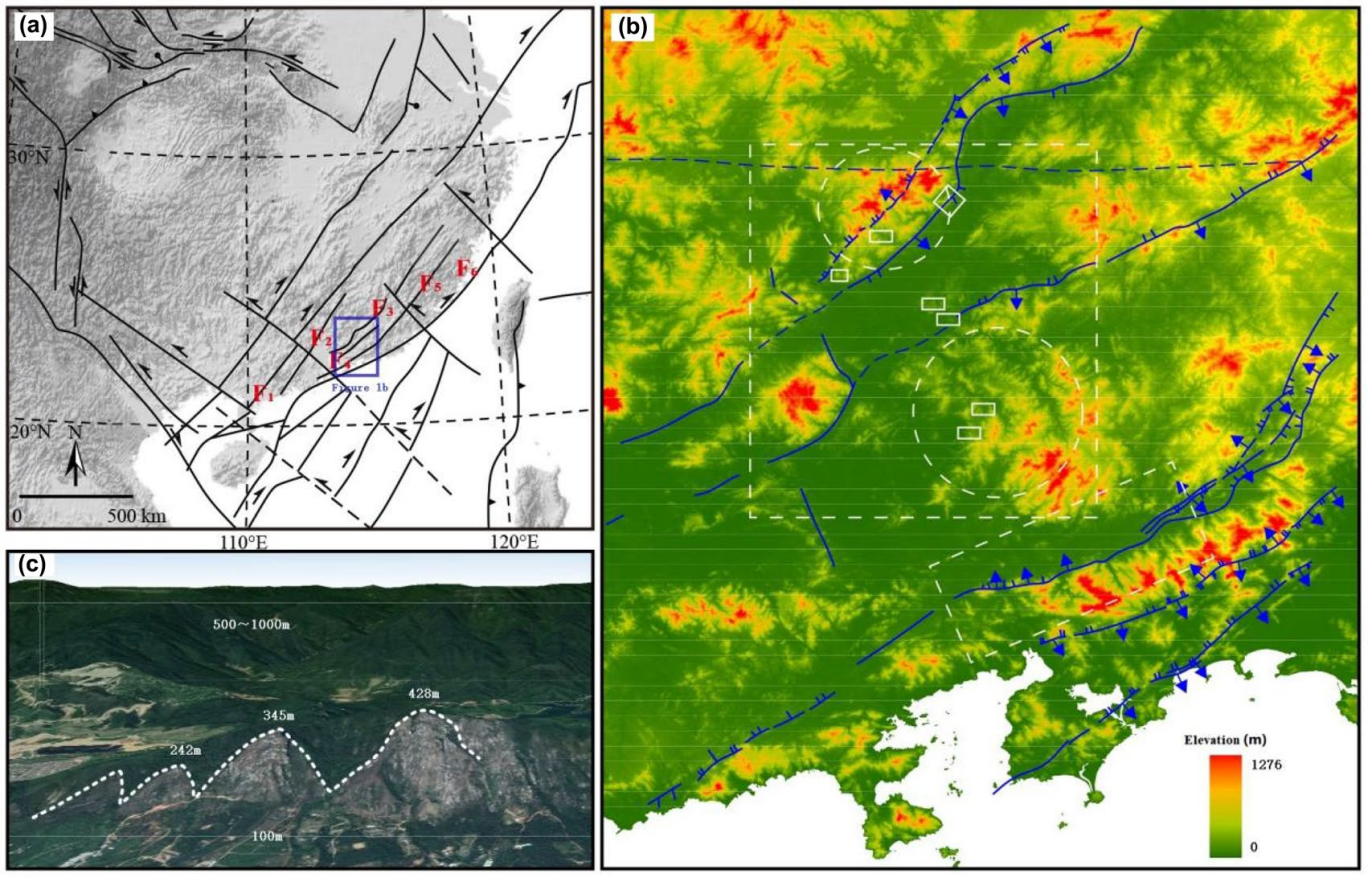
(**a**) Grotectonic map of the study area and adjacent areas; (**b**) topographic map of Huiyang–Meixian depression basin and its surrounding; (**c**) the triangle facet of Heyuan fault. F1.Wuchuan-Sihui Fracture; F2.Enping-Sinfeng Fracture; F3.Yangjiang-Heyuan Fracture; F4.Zijin-Boro Fracture; F5.Zhenghe-Dapu Fracture; F6.Changle-Nan'ao Fracture. According to Refs.^20^.

**Figure 4:**
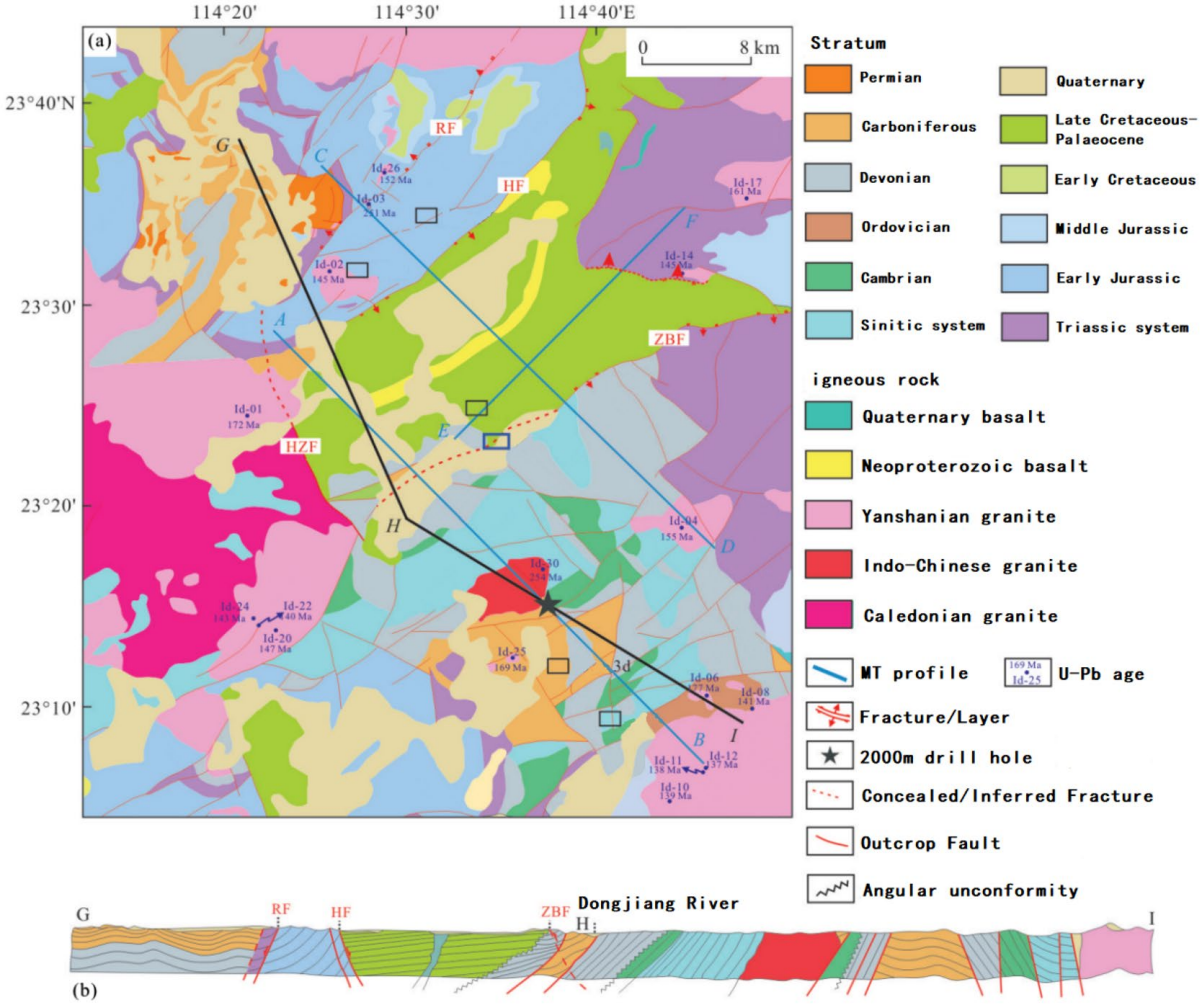
(**a**) Tectonic map of the study area and adjacent areas; (**b**) GHI profile in the study. RF. Herringbone Fracture; HF. Heyuan Fracture; HZF. Huizhou Fracture; ZBF. Zijin-Boro Fracture. According to Refs.^21^.

The study area exhibits well-developed fold and fault structures. The basement folds are represented by the Baishi Syncline, with a northeast-trending axis (Fig. 4a). The orientations of the overlying fold structures are predominantly northeast-trending, with subordinate east–west, northwest, and north–south trends. Representative examples include the Aibei compound syncline with a northeast-trending axis, the Shimen anticline with a northwest-trending axis, the Zhaoyuan syncline with an almost north–south-trending axis, and the Baoxi anticline with an approximately east–west-trending axis^20^. The dominant fault structures are oriented in the northeast direction, with a secondary northwest orientation, forming an overall concave–convex structural pattern (Figs. 3,  4b).The stratigraphic units exposed in the study area are similar to those exposed in the southern part of the Huaxia Massif^8^. Except for the Silurian strata, all other stratigraphic units are present in the study area (Fig. 4).

The study reveals the existence of two geothermal systems in the area: hydrothermal and dry hot geothermal resources. In terms of hydrothermal genesis, atmospheric precipitation, surface water, or ambient groundwater infiltrates through fault structures and weathered fractures. During the deep circulation process in the fractured zone, it extracts heat from normal or elevated rock temperatures and manifests at suitable locations through exposure due to uplift or human engineering activities. As for the dry hot genesis mechanism, the combined effect of high thermal conductivity and the presence of radioactive heat-generating elements in the granite body contributes to the formation of high-temperature geothermal resources in the study area. The granite body in the area is up to 3.5 km thick, predominantly composed of highly differentiated I-type granites from the Yanshanian period, especially those derived from remelting of crustal materials from the pre-Cambrian era, which contain high concentrations of heat-generating elements such as Th, U, and K. The radioactive decay of these heat-generating elements provides a significant amount of heat, thereby increasing the surface heat flow. Through comprehensive analysis, it is evident that the Moho surface in the study area is shallow, and the Moho temperature is relatively high, indicating a substantial contribution of mantle heat to the surface heat flow.

The data used in this study was obtained from the Guangdong Nonferrous Metal Geological Bureau and was utilized to create geological and topographic maps of the study area and its surrounding regional geology.

## Available geothermal energy, potential

The Guangdong–Hong Kong–Macao Greater Bay Area is rich in geothermal resources, and Huizhou City in particular has concentrated resources in Longmen County, Huicheng District, and Huidong County^21^.Currently, the utilization of geothermal resources in Huizhou City primarily focuses on hydrothermal geothermal energy, while the development and utilization of shallow geothermal energy and dry hot rock resources are still in the exploratory stage.

### Hydrothermal geothermal resources

Huizhou City, located in the eastern part of Guangdong Province, possesses multiple geothermal fields such as Boluo hot springs, Huidong Tangzi, and Huangshadong, displaying tremendous geothermal development potential^22,23^. In 2014, the Guangdong Geological Exploration Team conducted drilling in the Huangshadong area of Huizhou, providing new evidence for geothermal development. The hot springs in the study area are mainly distributed near Boluo and Huiyang, arranged in a northeast-southwest and northwest-southeast direction. Among them, the Huangshadong Geothermal Field in Shibai No. 1 is a renowned geothermal field in the region.

From a regional structural perspective, the geothermal fields are controlled by the superposition of larger-scale structures from the Yanshanian and more recent Xishanian periods. The presence of well-developed extensional faults and significant thermal alteration along rock contacts provides favorable pathways for the circulation of rock pore water, structural fracture water, and carbonate rock cave water, facilitating the upward flow of deep-seated heat. As a result, a large-scale hydrothermal geothermal field has formed in the region.

**Figure 5:**
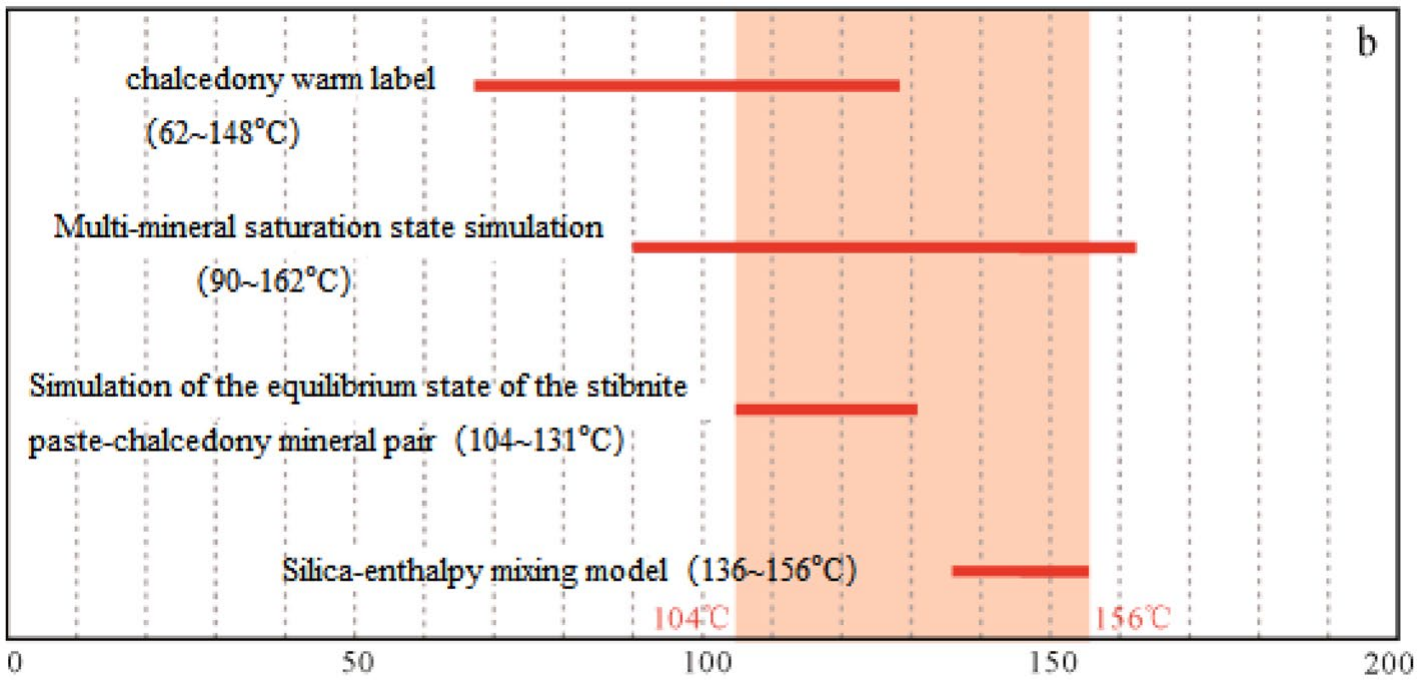
Coastal geothermal water reservoir temperature according to Refs.^24^.The red line is the temperature range.

The most likely temperature range for geothermal reservoirs in the Guangdong–Hong Kong–Macao Greater Bay Area is 104–156 °C. The circulation depths of geothermal water in inland and coastal areas can reach up to 4800 m and 4200 m, respectively^24^. Groundwater accounts for 84 °C of the circulating water in inland geothermal systems, while seawater contributes to 37% of the circulating water in coastal geothermal systems (Fig. 5).

### Hydrothermal geothermal resources

The geothermal resources in the Huangshadong area are related to granite. In this study, we conducted data collection and analysis specifically for the Huangshadong area, resulting in the following parameter Table 1.

**Table 1:** Calculating parameters of hot dry rock geothermal resources of granite in the study area.

Parameter	Huangshadong area	Shiba area
Density of granite(kg-m^3^)	2.73 × 10^3^	2.73 × 10^3^
Specific heat capacity of granite(kcal-kg^−1^-°C^-1^)	0.79	0.79
Density of underground hot water(kg-m^3^)	1 × 10^3^	1 × 10^3^
Specific heat capacity of groundwater(kcal-kg^1^-°C^−1^)	4.2	4.2
Porosity of granite (%)	0.5	0.5
Average temperature of granite and water (°C)	164	164
Local average annual temperature (°C)	20	20
Area of concealed rock body (km^2^)	18 × 13	22 × 12
Calculated thickness (km)	3.5	3.5
Q(kW-h)	8.07 × 10^14^	9.10 × 10^14^

After calculation, the annual heat production of granite in the Huangshadong working area is estimated to be 3.54 × 10^7^ kW h, while in the Shiba working area, it is estimated to be 4.62 × 10^7^ kW h. Assuming a conversion rate of 0.404 kg of standard coal per kilowatt-hour, it implies that the rock mass in the study area generates approximately 3.10 × 10^4^ tons of standard coal energy annually. Considering that the intrusion of the rock mass exceeds 137 million years, the cumulative heat production amounts to 4.38 × 10^12^ tons of standard coal energy. In 2021, the total energy consumption in Huizhou City was approximately 3 × 10^7^ tons of standard coal, which is equivalent to 1.46 × 10^5^ times the energy consumption in the same year^25,26^.

One notable distinction between the utilization of enhanced geothermal systems (EGS) for power generation and conventional thermal power plants lies in their respective heat extraction methods. EGS employ a closed-loop system that ensures the absence of wastewater, waste materials, and emissions, thereby mitigating any potential environmental impact associated with traditional thermal power generation methods^27^.

**Figure 6:**
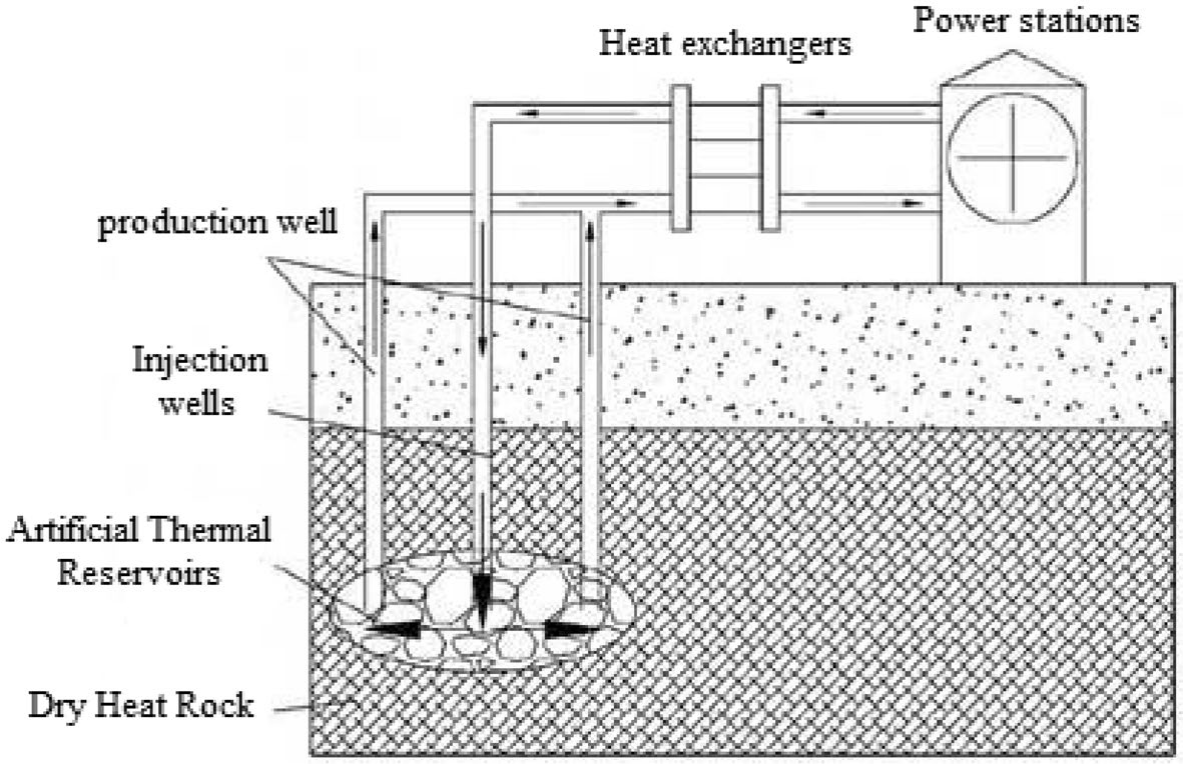
Enhanced geothermal system (EGS) power generation plant diagram.

The process of harnessing geothermal power through enhanced geothermal systems (EGS) entails injecting low-temperature water into the geothermal reservoir. Subsequently, as the water permeates through fractures and weathered fissures, it undergoes a deep circulation process within the fractured zone. During this process, the water absorbs heat from the surrounding rock mass, either at normal or elevated temperatures. Upon reaching suitable locations, the heated water ascends to the surface, either naturally exposed or artificially revealed through engineering interventions. It emerges as a mixture of high-temperature water and steam, which is then collected through production wells for electricity generation (Fig. 6).

## Energy use and economic analysis

**Figure 7:**
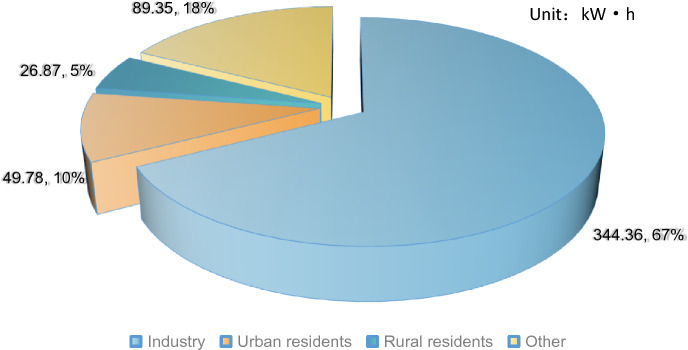
Distribution of annual social electricity consumption in Huizhou. Figureures are electricity consumption, percentages are shares.

According to the 《2022 Huizhou Statistical Yearbook》, the total electricity consumption in Huizhou City in 2021 was 51.036 billion kW h (the total electricity consumption in the Greater Bay Area exceeded 760 billion kW h, with Shenzhen’s electricity consumption reaching 110.34 billion kW h, representing a growth rate of 14.0%.Fig. 7 shows the values and shares of electricity consumption for each social component.

The power structure in the Huizhou region is primarily composed of fossil fuels and renewable energy. Fossil fuels, including coal, oil, and natural gas, account for approximately 80% of the total, while renewable energy, mainly consisting of hydropower, wind power, and solar energy, accounts for approximately 20%.

Although thermal power generation has low construction costs and short construction periods, allowing for adjustment based on load variations, it has significant drawbacks. The continuous emissions of gases such as SO_2_ and NO_2_ from coal combustion have led to increased acid rain in many regions of China, causing fly ash pollution near power plants and adversely affecting people's lives and plant growth. Thermal power plants typically use water as a cooling medium, with a daily water consumption of about 100,000 tons for a 1000MW thermal power plant. In 2021, Huizhou's total energy consumption reached 29.96 million tons of standard coal.

In Huizhou City, specifically within the Huangsha Cave study area of the industrial zone, there is an accumulated heat production of 4.38 × 10^12^ tons of standard coal energy. This quantity is equivalent to 1.46 × 10^5^ times the total energy consumption in Huizhou for the same period. Additionally, the rock formations within the study area generate a new quantity of 3.10 × 10^4^ tons of standard coal energy annually. Similar geothermal resource areas to the Huangsha Cave study area are widespread throughout the Greater Bay Area. If large-scale application of geothermal energy can be realized, it would positively impact the energy structure optimization of the entire Greater Bay Area.

## Sustainability assessment

Geothermal energy, as a clean and sustainable energy source, To maximize its sustainability and environmental advantages, several approaches can be taken:

1. Geothermal energy can supplement other renewable energy sources such as wind and solar power, providing stable and continuous electricity supply in situations where wind and solar energy are intermittent. By using a mix of renewable energy sources, the power grid can become more reliable and flexible.
2. Geothermal energy can help reduce dependence on fossil fuel-based electricity generation. By replacing coal-fired power plants with geothermal power stations, we can significantly reduce greenhouse gas emissions and air pollution.
3. Geothermal heat pumps can be used for heating and cooling buildings, leading to substantial energy savings and reductions in greenhouse gas emissions. This technology is applicable to residential, commercial, and industrial buildings.

The Greater Bay Area possesses favorable geological conditions for the development of geothermal energy. However, the current methods of development and utilization are inefficient, characterized by a low degree of resource exploration and a singular form of utilization. Geological surveys indicate a high geothermal gradient in the Greater Bay Area, which, coupled with the region's substantial energy demand, suggests that geothermal energy could play a pivotal role. The application of geothermal energy not only reduces air and water pollution but also creates employment opportunities and generates economic benefits. Most importantly, it alleviates the energy burden, holding significant implications for the sustainable development and energy structure optimization of the entire Greater Bay Area.

Therefore, the Chinese government should intensify exploration and assessment efforts regarding geothermal energy. It is imperative to understand the geological conditions, thermal storage characteristics, and quality and quantity of geothermal resources in the Greater Bay Area, specifically focusing on hydrothermal, shallow geothermal, and HDR regions. This comprehensive evaluation will serve as a foundation for the rational development and utilization of geothermal energy.

## Conclusion

1. Geothermal energy is an abundant, widely distributed, and reliable renewable energy source.It holds great significance in implementing the ecological civilization concept and realizing carbon peaking and carbon neutrality goals.
2. The further development of the Greater Bay Area is constrained by resource limitations and severe ecological environmental pressures. The issue of energy consumption and economic growth imbalance needs urgent attention. Geothermal energy presents an opportunity to mitigate the reliance on fossil fuels, reduce dependence on imported energy, and enhance national energy security. The exploration and utilization of geothermal resources become particularly pressing in this context. In the Huangsha Cave study area of Huizhou City, the cumulative geothermal energy amounts to 4.38 × 10^12^ tons of standard coal, equivalent to 1.46 × 10^5^ times the total annual energy consumption in Huizhou. Additionally, the rock formations in the study area generate an additional 3.10 × 10^4^ tons of standard coal energy per year. It is noteworthy that regions rich in geothermal resources, similar to the Huangsha Cave study area, are abundant in the Guangdong–Hong Kong–Macao Greater Bay Area. Large-scale utilization of geothermal energy in these regions is poised to have a positive impact on the overall energy structure optimization of the Greater Bay Area. The large-scale application of geothermal energy holds the potential to significantly enhance the structural efficiency of the energy system in the Greater Bay Area.
3. The Greater Bay Area possesses favorable geological conditions for the development of geothermal energy. However, the current methods of development and utilization are inefficient, characterized by a low degree of resource exploration and a singular form of utilization. According to geological surveys, Guangdong Province exhibits a high geothermal gradient, and the Greater Bay Area has substantial energy demand that can be met by geothermal energy. Therefore, there is considerable potential for the application of geothermal energy in the Greater Bay Area. The Chinese government should intensify exploration and assessment efforts regarding geothermal energy, focusing on understanding the geological conditions, thermal storage characteristics, and quality and quantity of geothermal resources in Guangdong Province, specifically in hydrothermal, shallow geothermal, and HDR regions. This comprehensive evaluation will provide a basis for the rational development and utilization of geothermal energy.
